# Acute Ischemic Stroke With Central Retinal Artery Occlusion as a Rare Presentation of COVID-19 Disease

**DOI:** 10.7759/cureus.17469

**Published:** 2021-08-26

**Authors:** S.K. Jakaria Been Sayeed, Subir Chandra Das, Reaz Mahmud, Md Moniruzzaman, Md Mujibur Rahman

**Affiliations:** 1 Medicine, National Institute of Neurosciences and Hospital, Dhaka, BGD; 2 Clinical Neurology, National Institute of Neurosciences and Hospital, Dhaka, BGD; 3 Neurology, Dhaka Medical College Hospital, Dhaka, BGD; 4 Medicine, Bangabandhu Sheikh Mujib Medical University, Dhaka, BGD

**Keywords:** central retinal artery occlusion, ischemic stroke, covid-19, rare presentation, case report

## Abstract

In this report, we present a case where the patient developed a border-zone ischemic stroke with central retinal artery occlusion (RAO) following coronavirus disease 2019 (COVID-19) disease. The COVID-19 disease has been described to induce inflammatory changes that predispose to thrombotic disease in both venous and arterial circulation. Angiotensin-converting enzyme 2 (ACE2) receptor expression in the blood vessel with which severe acute respiratory syndrome coronavirus 2 (SARS-CoV-2) binds is the cornerstone of inflammation although the pathogenesis of central RAO is multifactorial. The effects of COVID-19 inflammatory and pro-coagulant state on cerebral and retinal vascular systems are still inadequately understood. Combined presentation of central RAO with ischemic stroke has not been documented in the literature yet. As of now, no guidelines exist regarding treatment modalities to be employed in such instances. Hence, further research is warranted regarding the treatment of this condition with respect to the association with COVID-19.

## Introduction

The World Health Organization (WHO) has declared coronavirus disease 2019 (COVID-19) as a pandemic since March 2020. Primarily, COVID-19 patients presented with features of respiratory tract infection. But it can present as a multisystemic disease involving gastrointestinal, cardiac, renal, vascular, and neurologic systems. Sole neurological manifestations like headache, confusional state, cerebrovascular disease such as ischemic or hemorrhagic stroke, sometimes with a branch or central retinal artery occlusion (RAO), are reported infrequently [1,2]. The pathogenesis of stroke or RAO in COVID-19 is multifactorial. The Inflammatory and hypercoagulable states are also established risk factors for this thrombotic event causing large to medium artery occlusion [3,4]. However, there have been very few reports on central or branched RAO in COVID-19. Reports on combined ischemic stroke and central RAO associated with COVID-19 are still to be documented. In this report, we discuss a case of concomitant ischemic stroke and central branched RAO following severe acute respiratory syndrome coronavirus 2 (SARS-CoV-2) infection.

The informed consent form has been attached as a supplementary file in the Appendix.

## Case presentation

**Figure 1:**
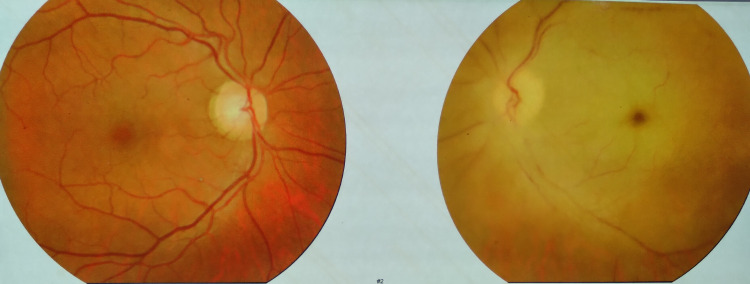
Fundoscopic examination The optic disc was pale with chalky white discoloration with a cherry-red spot in the macula of the temporal part of the left eye. The vessel count was reduced

The patient was a 38-year-old male, who was diabetic, nonsmoker, normotensive, and resident of a divisional city in Bangladesh. He was hospitalized with a four-day history of fever, dry cough, and progressive dyspnea in a COVID-19-dedicated unit of a tertiary care hospital in the respective divisional city. He tested positive for reverse transcription-polymerase chain reaction (RT-PCR) for COVID-19. On admission, his pulse was 110 per minute, regular, blood pressure was 120/80 mmHg, respiratory rate was 24 breaths per minute, and his SpO_2_ was 92% on room air. He was receiving standard treatment for COVID-19. His oxygen saturation was maintained with four liters of supplemental oxygen. On the seventh day of his admission, he developed right-sided hemiparesis with the blurring of vision on the left eye. After RT-PCR for COVID-19 being negative 14 days after the initial report, he was referred to a super-specialty hospital in Dhaka, the capital city, for treatment and further evaluation. On admission, the patient was found to be slightly disoriented with a Glasgow Coma Scale (GCS) score of 14/15; he had right-sided hemiparesis with a muscle power of 2/5 with extensor plantar response. Visual acuity on the right was 6/6, and on the left, it was limited to finger counting from 1 meter. Projection of light and that of ray were present, and direct light reflex was sluggish with normal consensual reflex in the left eye. Intraocular pressure as measured by tonometer in both eyes was 15 mmHg. Fundoscopic examination revealed a mildly dilated left pupil with clear media. The optic disc was pale with chalky white discoloration with a cherry-red spot in the macula. The vessel count was reduced (Figure 1).

The right eye, optic disc, and retina were found normal. Due to the increasing requirement of supplemental oxygen (10 L per minute with nonrebreathing mask), the patient was shifted to a high-dependency COVID-19 unit and treated with high-flow oxygen, injectable anticoagulant, dexamethasone, and remdesivir according to the national guideline on COVID-19 management. Blood tests revealed elevated inflammatory markers with a procoagulant state (Table 1). 

**Table 1:** Laboratory value of the patient during admission ALT: alanine aminotransferase; APTT: activated partial thromboplastin time; ANA: antinuclear antibody; ANCA: anti-neutrophilic cytoplasmic antibody; CRP: c-reactive protein; LDH: lactate dehydrogenase; Na: sodium; K: potassium

Marker	Level	Reference
Hemoglobin	12.5	13-17 g/dl
White blood cell	18,000	4-11 × 10^3^/mm^3^
Neutrophil	16,000	2-7.5 × 10^3^/mm^3^
Lymphocyte	1,260	1.5-4 × 10^3^/mm^3^
Platelet	411,000	150-450 × 10^3^/mm^3^
ESR	58	0-12 mm
CRP	253	<10 mg/L
Serum ferritin	621.34	20-300 ng/ml
Serum LDH	464	134-214 IU/L
D-dimer	10	<0.50 µg/ml
ALT	72	10-40 IU/L
Random blood sugar	4.67	3.5-7.8 mmol/l
HbA1C	10.3	3.5-5.7%
Serum creatinine	0.75	0.72-1.25 mg/dl
Prothrombin time	13	11-16 seconds
APTT	41	26-38 seconds
p and c ANCA	Negative	
ANA	0.382	<1.2
Serum homocysteine	8.1	Adults: <18 µmol/L
Serum Na	126	135-145 mmol/l
Serum K	4.5	3.5-4.5 mmol/l
Serum chloride	95	95-105 mmol/l
RT-PCR for COVID-19	Positive	

**Figure 2:**
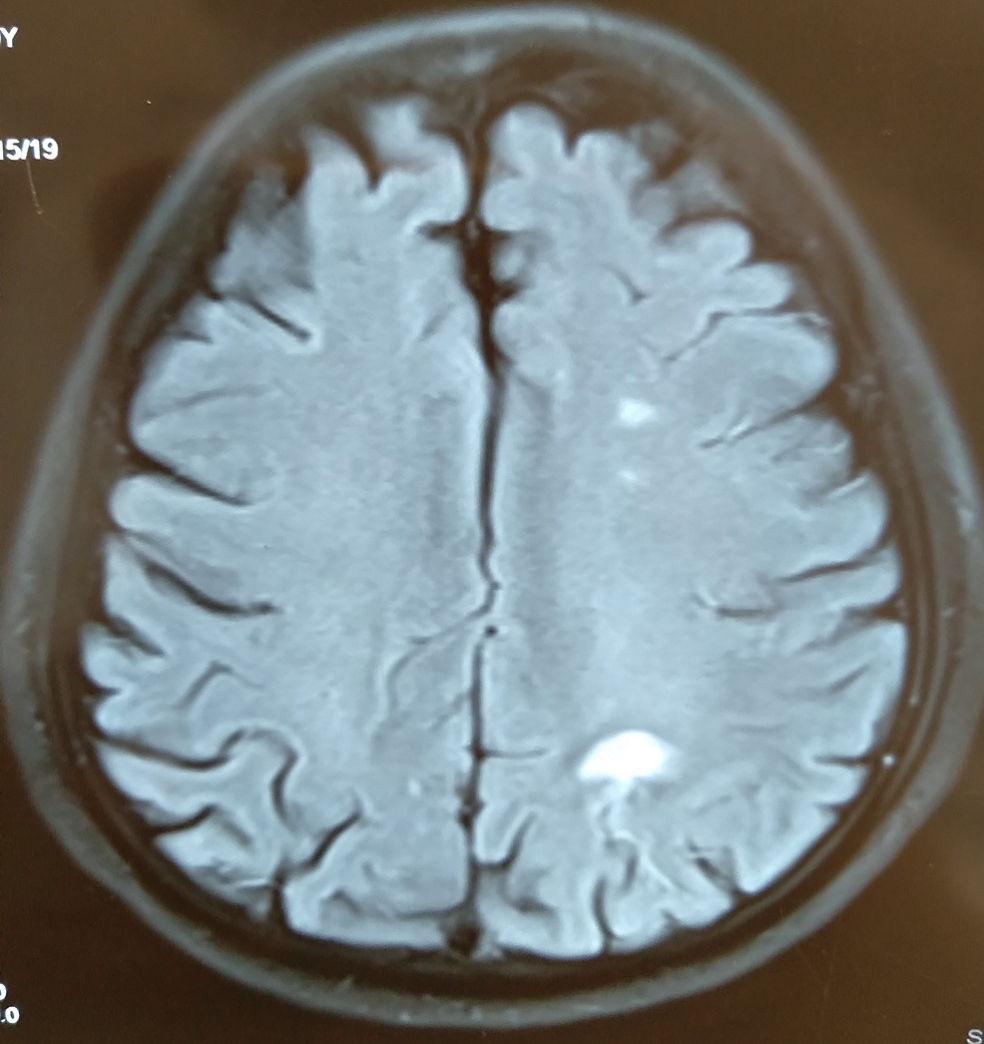
MRI of the brain with DWI The image shows an acute infarct in the border zone of the carotid and vertebral artery region in the left parieto-occipital region DWI: diffusion-weighted imaging; MRI: magnetic resonance imaging

MRI of the brain with DWI image showed acute infarct in the border zone of the carotid and vertebral artery region in the left parieto-occipital region (Figure 2).

**Figure 3:**
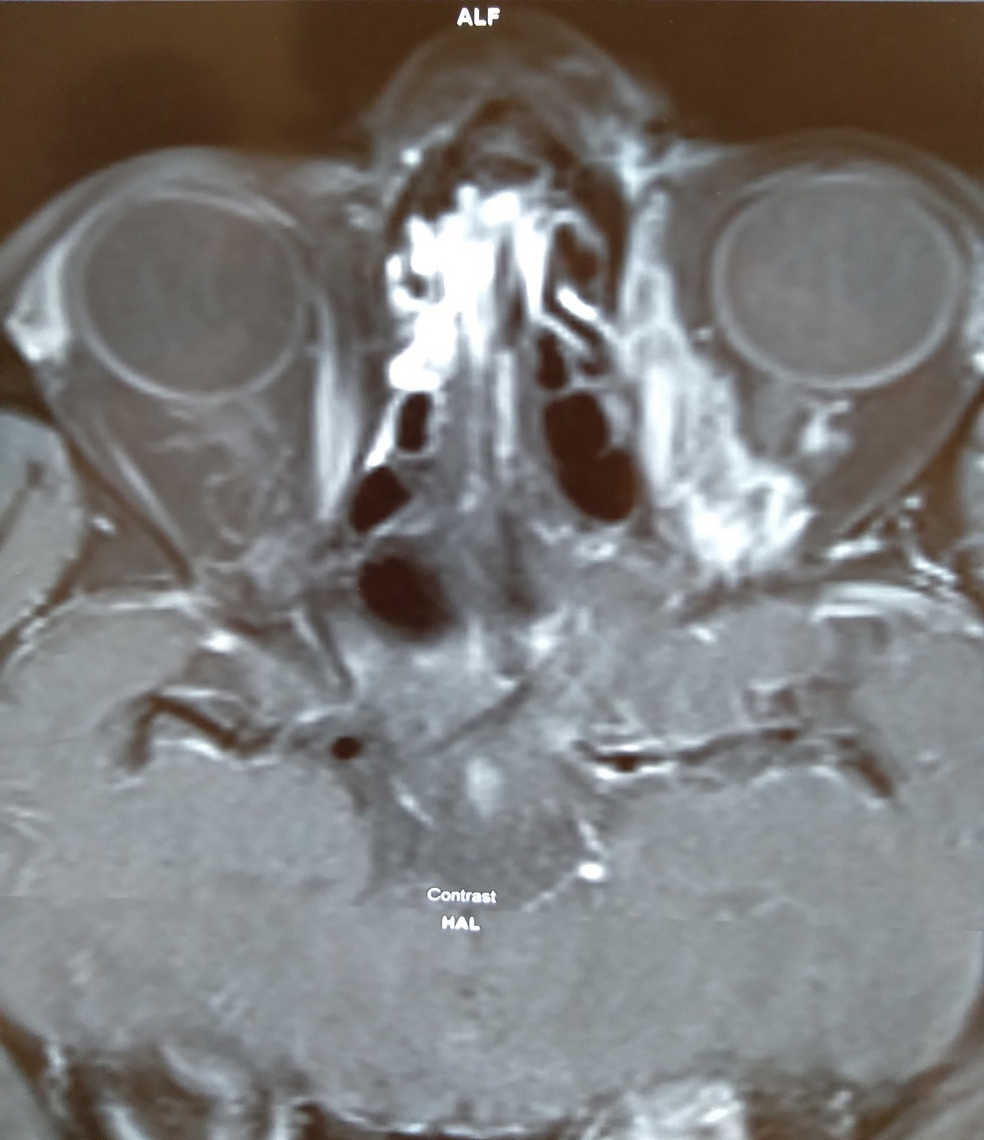
MRI of orbit revealed swollen left optic nerve and capillary hemangioma in the retrobulbar region MRI: magnetic resonance imaging

MRI of orbit revealed swollen left optic nerve and capillary hemangioma in the retrobulbar region (Figure 3).

**Figure 4:**
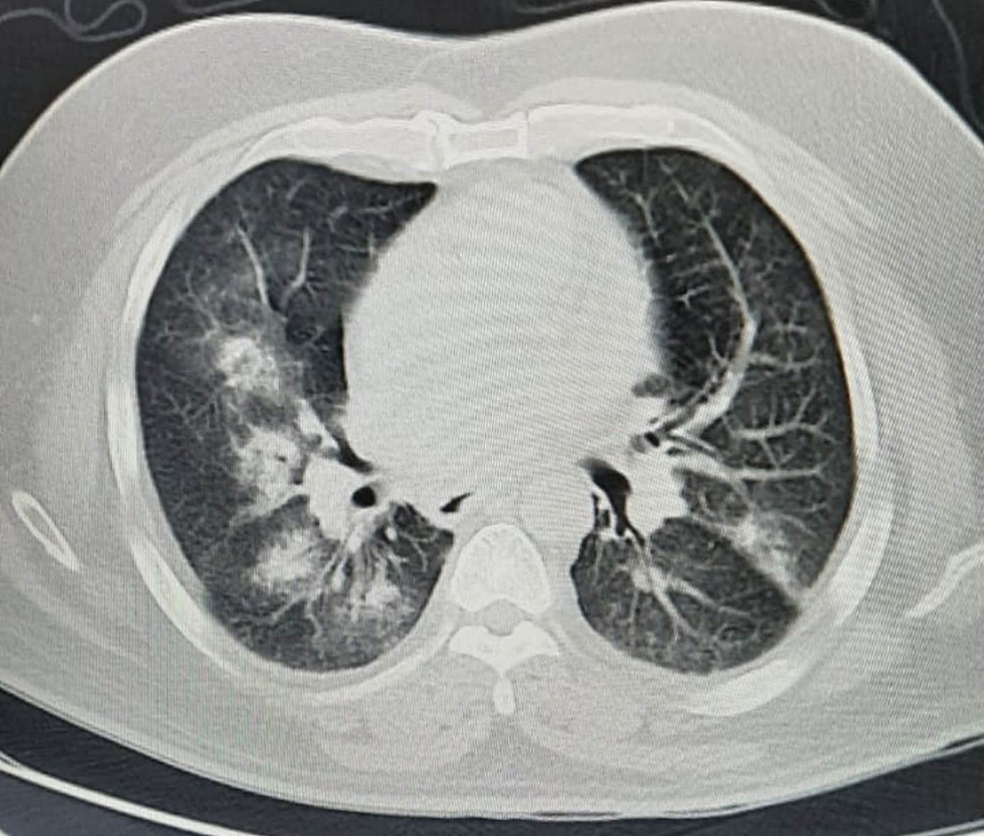
HRCT chest showed extensive bilateral ground-glass opacities HRCT: high-resolution computed tomography

A high-resolution CT chest showed extensive bilateral ground-glass opacities (Figure 4).

A bedside echocardiogram was done, which was found normal. We could not perform a carotid angiogram or carotid duplex due to the patient's deteriorating condition. Four days later, the patient's condition deteriorated further and he was transferred to the ICU as SpO_2_ was persistently falling lower than 70% despite high-flow oxygen supplementation (40 L per minute). In the ICU, he was intubated to receive mechanical ventilation and other supportive treatment. Despite all the efforts, the patient died after three days of ICU admission.

## Discussion

The COVID-19 disease has been described to induce inflammation-induced homeostasis changes that predispose to thrombotic disease in both venous and arterial circulation [3,4]. The risk of thromboembolic events in COVID-19 patients is currently under investigation, with preliminary results showing significantly prolonged prothrombin time, high D-dimer levels, and increased concentrations of proinflammatory cytokines and biomarkers of inflammation in patients with more severe disease, indicating the likelihood of disseminated intravascular coagulation or thrombotic microangiopathy [4]. In the present case, the patient developed a border-zone ischemic stroke with central RAO after suffering from severe COVID-19 disease. It seemed to be a thrombus in the left internal carotid artery with an embolic phenomenon in the left ophthalmic artery. Central RAO can occur due to vasculitides like giant cell arteritis and atherosclerosis [5]. Blood vessels contain angiotensin-converting enzyme (ACE) receptors for SARS-CoV-2; binding of the virus with the receptor of the endothelium may induce a local inflammatory change, which predisposes to the formation of the thrombus. Retinal vascular damage could also be correlated to the ocular expression of the ACE2 and the interaction of SARS-CoV-2 with these receptors [6].

Systemic response in relation to viral infection might also play a role in the forming of thrombus at different sites [7]; Grau et al. [8] found that recent viral or bacterial infection significantly increased the risk of cardioembolism and arterio-arterial embolism. To our knowledge, a few cases have been reported, by Montesel et al. [2], Acharya et al. [9], and Dumitrascu et al. [10], where patients developed central RAO in association with COVID-19 disease. However, ischemic stroke associated with COVID-19 has been reported already [11]. It is the first case where a patient unfortunately simultaneously developed ischemic stroke and central RAO following severe COVID-19 disease. In the first reported case, central RAO developed during the second week of the patient’s hospitalization [9]. In the third case, reported by Dumitrascu et al. [10], the onset of the central RAO developed during the sixth week. In both cases, only fundal findings were reported. In the first case, reported by Montesel et al. [2], the interval between the disease onset and the development of central RAO was not reported. Nevertheless, in this instance, the patient developed ischemic stroke and central RAO on the fourth day of hospitalization, the seventh day of symptoms' onset. It is worthy to mention that our patient did not have hypertension or heart disease or other comorbidities except diabetes; he had not received any medications or COVID-19 vaccine, which might cause thrombotic events. Unfortunately, our patient passed away on day 23 due to possible sepsis with multi-organ failure. One limitation of this report is that we could not perform fluorescence fundal and magnetic resonance angiography as the patient was unstable, semiconscious, and was in ventilation. Nevertheless, this is the first case to be reported of a patient with central RAO with ischemic stroke following SARS-CoV-2 infection. We hypothesize that the inflammatory and pro-coagulant state secondary to COVID-19 can trigger retinal artery and internal carotid artery thrombosis. The time window for the intervention in the case of central RAO is four hours [12]. Though our patient was receiving subcutaneous heparin, it did not prevent the development of the thrombus. There is still no recommendation or guideline regarding the use of thrombolysis in such instances.

## Conclusions

The association between thrombosis and COVID-19 is becoming clear day by day. However, the effects of COVID-19 inflammatory and pro-coagulant state on cerebral and retinal vascular systems are still inadequately understood. There are still no guidelines regarding treatment modalities to be employed in such instances. Hence, further research is warranted regarding the treatment of this condition with respect to the association with COVID-19.

## Figures and Tables

**Figure 5:**
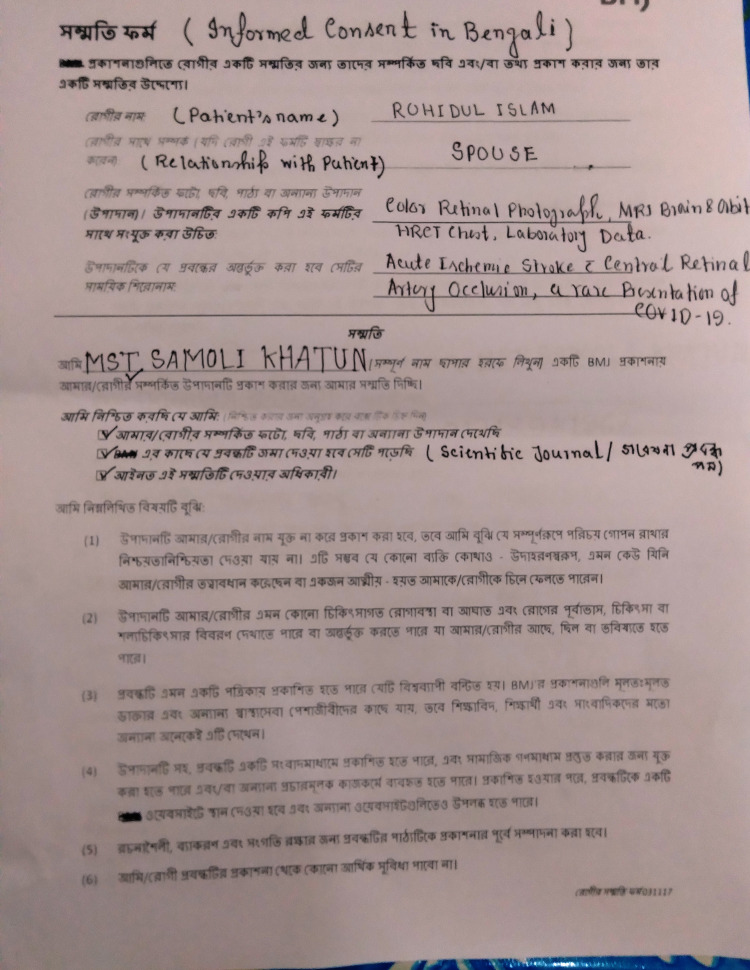
Informed consent in Bengali - page 1

**Figure 6:**
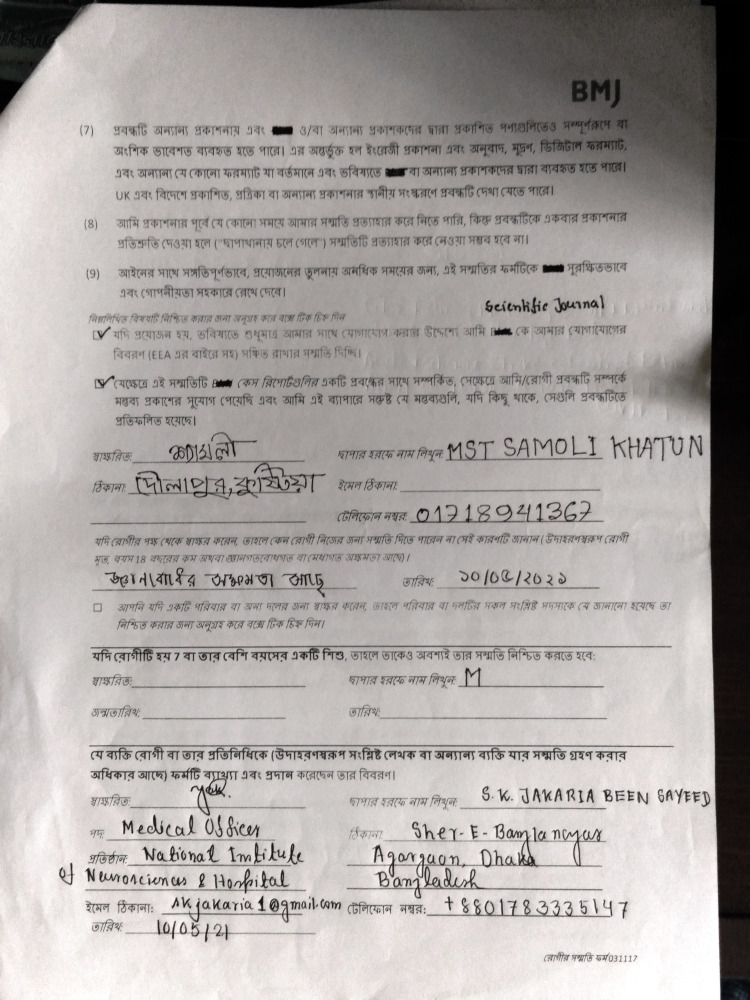
Informed consent - page 2
